# Genomic selection for survival under naturally occurring *Saprolegnia* oomycete infection in farmed European whitefish *Coregonus lavaretus*

**DOI:** 10.1093/jas/skad333

**Published:** 2023-10-01

**Authors:** Federico C F Calboli, Terhi Iso-Touru, Oliver Bitz, Daniel Fischer, Antti Nousiainen, Heikki Koskinen, Miika Tapio, Ilma Tapio, Antti Kause

**Affiliations:** Natural Resources Institute Finland (LUKE), FI-31600 Jokioinen, Finland; Natural Resources Institute Finland (LUKE), FI-31600 Jokioinen, Finland; Natural Resources Institute Finland (LUKE), FI-31600 Jokioinen, Finland; Natural Resources Institute Finland (LUKE), FI-31600 Jokioinen, Finland; Natural Resources Institute Finland (LUKE), FI-70210 Kuopio, Finland; Natural Resources Institute Finland (LUKE), FI-70210 Kuopio, Finland; Natural Resources Institute Finland (LUKE), FI-31600 Jokioinen, Finland; Natural Resources Institute Finland (LUKE), FI-31600 Jokioinen, Finland; Natural Resources Institute Finland (LUKE), FI-31600 Jokioinen, Finland

**Keywords:** ddRAD, genomic selection, infection resistance, *Saprolegnia*, whitefish

## Abstract

*Saprolegnia* oomycete infection causes serious economic losses and reduces fish health in aquaculture. Genomic selection based on thousands of DNA markers is a powerful tool to improve fish traits in selective breeding programs. Our goal was to develop a single nucleotide polymorphism (**SNP**) marker panel and to test its use in genomic selection for improved survival against *Saprolegnia* infection in European whitefish *Coregonus lavaretus*, the second most important farmed fish species in Finland. We used a double digest restriction site associated DNA (ddRAD) genotyping by sequencing method to produce a SNP panel, and we tested it analyzing data from a cohort of 1,335 fish, which were measured at different times for mortality to *Saprolegnia* oomycete infection and weight traits. We calculated the genetic relationship matrix (**GRM**) from the genome-wide genetic data, integrating it in multivariate mixed models used for the estimation of variance components and genomic breeding values (**GEBVs**), and to carry out Genome-Wide Association Studies for the presence of quantitative trait loci (**QTL**) affecting the phenotypes in analysis. We identified one major QTL on chromosome 6 affecting mortality to *Saprolegnia* infection, explaining 7.7% to 51.3% of genetic variance, and a QTL for weight on chromosome 4, explaining 1.8% to 5.4% of genetic variance. Heritability for mortality was 0.20 to 0.43 on the liability scale, and heritability for weight was 0.44 to 0.53. The QTL for mortality showed an additive allelic effect. We tested whether integrating the QTL for mortality as a fixed factor, together with a new GRM calculated excluding the QTL from the genetic data, would improve the accuracy estimation of GEBVs. This test was done through a cross-validation approach, which indicated that the inclusion of the QTL increased the mean accuracy of the GEBVs by 0.28 points, from 0.33 to 0.61, relative to the use of full GRM only. The area under the curve of the receiver–operator curve for mortality increased from 0.58 to 0.67 when the QTL was included in the model. The inclusion of the QTL as a fixed effect in the model increased the correlation between the GEBVs of early mortality with the late mortality, compared to a model that did not include the QTL. These results validate the usability of the produced SNP panel for genomic selection in European whitefish and highlight the opportunity for modeling QTLs in genomic evaluation of mortality due to *Saprolegnia* infection.

## Teaser

We demonstrate the successful use of Next Generation Sequencing for genomic selection in aquaculture, testing survival to *Saprolegnia* infection, a disease-causing serious economic losses in aquaculture. We identify a strong QTL affecting survival, and we show that including the QTL in an enhanced BLUP approach improves the estimation of breeding values.

## Introduction

The advent of high throughput genetic technologies has allowed to genotype animals for hundreds, thousands, or even millions of different DNA markers, and to integrate this data in breeding programs. The availability of genome-wide data has allowed to extension of the genetic-based selection techniques, such as marker-assisted selection (**MAS**; Lande and Thomson, 1990; You et al., 2020) to more powerful approaches, such as genomic selection (Meuwissen et al., 2001; Goddard and Hayes 2007). Genomic evaluation with tens of thousands of DNA markers has been proven to be an effective selective tool in animal breeding because quantitative traits are typically determined by multiple genes with minor effects on the phenotype. Hence, MAS that specifically modeled only a few major quantitative trait loci (**QTL**) of quantitative traits in a breeding value evaluation has been in fact largely abandoned in practice (Misztal et al., 2020).

For aquaculture species, where the domestication period is short (Houston et al., 2020), the presence of QTL of large effect is surprisingly common for disease resistance traits (Fuji et al., 2007; Moen et al., 2009; Houston et al., 2010; Aslam et al., 2020; Karami et al., 2020; Calboli et al., 2022, Fraslin et al., 2022a; Vallejo et al 2022; Vela-Avitúa et al., 2022, 2023). This observation and all the instances where QTLs of large effects have been observed have induced a renewed interest in the explicit integration of QTL information in genetic evaluations (Li et al., 2017; Lopes et al., 2017; Nani et al., 2019; Ren et al., 2021; Fraslin et al., 2022a).

*Saprolegnia* is a naturally occurring oomycete pathogen affecting aquaculture species, causing large losses, especially in freshwater growing conditions (Van Den Berg et al., 2013). At present, work has been carried out to identify the presence of genetically different *Saprolegnia* strains (Sarowar et al., 2019) in different hosts and growing conditions (Sandoval-Sierra et al., 2014), but far less information is available on the genetic resistance to *Saprolegnia* infection in host species (Misk et al., 2022).

Our aim in this work was manyfold. First, we aimed to test the feasibility of using next generation sequencing (**NGS**) to identify a novel panel of single nucleotide polymorphisms (**SNPs**) for genomic selection for commercial traits in whitefish, an aquaculture species that currently lacks commercial/off-the-shelf genetic marker chips that can be used for selection. We developed a SNP panel for European whitefish (*Coregonus lavaretus* L.) through double digest restriction site associated DNA (ddRAD) sequencing, using the recently published reference genome (De-Kayne et al., 2020). Secondly, we aimed to understand the genetic of mortality *Saprolegnia* infection, a common, commercially relevant infection. We were especially interested in understanding whether the genetic architecture of resistance is completely polygenic, or is based on one or a few QTLs explaining most of the genetic variance, or a mixture of these architectures. We genotyped a cohort of fish kept in freshwater tanks in the Natural Resources Institute Finland’s breeding program, and we followed their mortality due to *Saprolegnia* infection to sexual maturity. Finally, we were interested in whether an NGS-based genomic evaluation would provide accurate genomic estimated breeding values (**GEBVs**) and whether the explicit modeling of any major QTL would be useful in genomic selection-based breeding programs.

## Materials and Methods

### Animal care

The establishment of progeny families at Natural Resources Institute Finland’s fish breeding facilities followed the protocols approved by the Natural Resources Institute Finland’s Animal Care Committee, Helsinki, Finland.

### Fish stock

We analyzed data from the Natural Resources Institute Finland’s breeding program, housed in freshwater facilities at Enonkoski research station. *Saprolegnia* is present in the water source and thus fish were naturally exposed to the pathogen. In November 2018, a cross was carried out between 26 sires and 100 dams that resulted in a cohort of 1,671 fish (all offspring of the same age), whose data we present here. Mating was carried out for 1 wk, and the eggs from all mating were pooled after the fertilization. The eggs were incubated in a single large incubator. At eyed stage, the eggs were placed in a single indoor tank in which the hatching occurred. These fish are the safe reserve of the main breeding program stock in which the families are typically incubated separately, reared separately in family tanks, individually tagged without a need for genotyping and only then pooled together (Kause et al., 2011).

### Phenotype data collection

Between January 18 and February 1, 2021, a total of 1,671 fish were individually tagged with passive integrated transponders (Biomark GPT12 pit tags) and a fin clip was sampled for genotyping, and the fish were then moved to an outdoor tank to initiate the experiment. The tank was an concreate, flow through tank of size of 63 m^2^. The farm uses water from an upstream lake, and *Saprolegnia* occurs naturally in this catchment area and has caused disease outbreaks annually in recent years. At tagging, all fish were weighted – this weight is the “Weight_2_” trait in our data. The choice of numbering follows the growth seasons, hence “Weight_2_” is the weight at the second growth season. Following tagging, mortality to *Saprolegnia* infection was collected daily during weekdays. A dying fish with the growth of fungus on the surface of a fish was captured with a hand net and its id-tag was recorded. If fish died and it did not have fungal growth on it, it was not considered to be dying due to *Saprolegnia* (Table 1).

**Table 1.** Traits summary. Sample size and mean values for recorded traits, and number of fish alive at measuring date, number of fish found dead to *Saprolegnia* up to a measuring date, and number of missing fish. Because some fish were lost (likely due to predation) the sample size indicates the actual number of fish that could be scored for mortality at any given time

	Sample size	Mean			
Weight_2_ (g)	1,335	279.87			
Weight_3_ (g)	620	756.2			
Height/Length_3_ (dimensionless ratio)	620	0.23			

	Sample size	Incidence	# of fish alive	# of fish dead due to fungus	# of fish missing
Mortality_3_ (count)	836	0.31	576	260	499
Mortality_4_ (count)	473	0.52	228	245	103
Mortality_tot_ (count)	733	0.69	228	505	602

On May 11, 2022, all fish were measured for body weight “Weight_3_” and body height to body length ratio “Height/Length_3_”. Height/Length_3_ is one of the selected traits in the breeding program, because an elongated shape is preferred (Kause et al., 2011). Mortality due to *Saprolegnia* up to that day is termed “Mortality_3_”. Mortality to *Saprolegnia* were coded as 1 and survival was coded as 0. Mortality to *Saprolegnia* infection was again collected as observed until November 8, 2022—mortality from time 3 up to that day is the “Mortality_4_” trait in our data. Mortality to *Saprolegnia* between time 3 and time 4 was coded as 1 and survival up to time 4 was coded as 0. Finally, we created a synthetic trait, Mortality_tot_, as the sum of Mortality_3_ and Mortality_4_. All the fish that died due to *Saprolegnia* during the study were coded as 1 and the fish that survived until time 4 were coded as 0. The fish that were not recaptured after tagging were coded as missing observations for mortality. Fish disappear because of predators such otters and herons.

### ddRAD library preparation

Adapting the protocol of Sala-Lizana and Oono (2018), DNA was extracted from the finclips using DNeasy 96 Blood & Tissue kit (Qiagen) following the manufacturer’s protocol. DNA concentration was measured by Nanodrop and normalized to 30 ng/µL concentration. About 500 ng of high-quality DNA per sample (17 µL; 30 ng/µL) was double-digested with two rare cutting restriction enzymes EcoRI-HF and SphI. The restriction was done in a volume of 20 to 17 µL DNA, 0.1 µL EcoRI-HF, SphI0.5 µL, 2 µL cutsmart buffer (10×), and 0.4 µL of molecular grade water at 37 °C for 15 min or 4 h, following heat-inactivating for 15 min at 65 °C. The subsequent ligation (add 2.5 µL ligation buffer, 1 µL of each adapter (100 nM), and 0.5 µL T4 ligase (NEB, New England, USA) of non-barcoded adapter was done at 16 °C for 14 min, following heat-inactivation at 65 °C for 15 min. Not incorporated adapters and other small DNA-fragments were purified/removed using SPRIselect magnetic beads (Beckman Coulter). First, the volume of each sample was adjusted with molecular-grade water to 50 µL and then 40 µL of magnetic beads were added (0.8× to select for fragments longer than 200 bp) following the manufacturer protocol. The purified DNA was resuspended in 25 µL of molecular-grade water. The quantity of each DNA was measured using the Qubitflex dsDNA HS (high sensitive) assay. Each sample was individually barcoded with the Illumina Nextera v2 combinatorial dual-indexed barcodes (i7 and i5). The indices were attached with 18 cycles of PCR using Phusion polymerase (Fisher Scientific) in 20 µL volume and an optimized annealing temperature of 61 °C. The amount of template was 5 µL. Subsequently, the PCR-products were quantified using Qubitflex with a dsDNA HS assay. Only products with a significantly higher amount than the NTC (no template control) were used for sequencing >3 ng/µL. Sample with a low amount of product was repeated if applicable with 1 or 2 more cycles.

### Sequencing

Single libraries were pooled in equimolar amounts and run on a 1.5% TAE gel. Fragments between 400 and 700 bps were cut out of the gel and purified with Qiagen Gel extraction Kit. The quality and size of the final, pooled sequencing library were checked on the Bioanalyzer or TapeStation using DNA HS assays. For sequencing the pooled library was adjusted to a concentration of 1 to 4 nM. Following the guidelines from Illumina, the libraries were diluted to a final concentration of 2.0 pM. The paired-end sequencing (2 × 150 bp) was done on the NextSeq 550 (Illumina).

Basecalling and demultiplexing were done with Illumina bcl2fastq2 Conversion Software (Illumina) with one index mismatch allowed. For each sample individually, the paired-end fragments were aligned to the reference genome (De-Kayne et al., 2020) using Bowtie (Langmead and Salzberg, 2012), retaining only those fragments that had a unique match to the genome. The selected fragments were sorted with Samtools (Li et al., 2009), which was used to generate a population-wide consensus call for each location of the genome covered by individual sample fragments. The genotyping by sequencing (**GBS**) pipeline was implemented in Snakemake (Mölder et al., 2021) and is an extension of the GBS SNP-Calling Reference Optional Pipeline (GBS-SNP-CROP) (Melo et al., 2016). This pipeline is publicly available (Fischer, 2023).

The raw ddRAD genotyping produced 65,318 SNPs. Following De-Kayne (2022), and after analysis of our data ­coverage patterns, all SNPs mapping to poor-quality reference assembly chromosomes 22, 28, 32, 35, 38, and 40 were removed. Using a quality control of at least 5% minor allele frequency, and a threshold of 80% genotyping success, 5,242 high-quality SNPs remained for 1,671 samples. Samples missing 50% or more of the genotypes of their SNPs were also removed, leaving us with a final dataset of 1,335 samples genotyped for 5,242 SNPs. In all analyses, each SNP genotype was coded as the standard 0/1/2 to reflect the number of minor alleles in the genotype (0, homozygote for the major allele; 1, heterozygote; 2, homozygote for the rare allele). In analyses where genotype (for any SNP) was used as a fixed effect in a liner model we transformed the 0/1/2 value as 1/2/3 to use genotype as a qualitative parameter with 3 levels with the software DMU (Madsen et al., 2014; see below).

### Estimation of heritability and genetic correlations

Phenotypic, genetic, and residual (co)variances of fish traits were estimated using the Average Information Restricted Maximum Likelihood module of *DMU* (DMUAI; Madsen et al., 2014). Heritabilities were estimated with a 2-trait multivariate model in which Weight_2_ was always included. Weight_2_ was available for all fish, whereas the other traits had some missing data, and hence this approach accounts for potential selection bias (Henderson, 1975; Ouweltje et al., 1988). Correlations were estimated with a series of 3-trait analyses. The model used for all traits was:


$$\begin{array}{l} Model\;1:\;y_{ij}=\;\mu_{j}+\;a_{ij}+\;e_{ij} \end{array}$$

in which *y*_*ij*_ is the phenotype of a trait *j* for the *i*th individual, µ_*j*_ is the mean value of a trait *j, a*_*ij*_ is the random genetic effect explained by the realized genomic relationship matrix between genotyped individuals (genetic relationship matrix [**GRM**]), and *e*_*ij*_ the random residual error. The GRM was calculated using *htginv* module of the MiX99 software package (Stranden and Lindauer, 1999) on the full SNP dataset of the offspring fish (‘full GRM’), using the first ­VanRaden method (VanRaden et al., 2009), without the need of using any correction to obtain an invertible matrix. Missing genotypes were imputed by *htginv*. Heritability was computed as:


$$\begin{array}{l} h^{2}={\sigma^{2}}_{G}/({\sigma^{2}}_{G}+{\sigma^{2}}_{E}) \end{array}$$

with *h*^*2*^ the heritability, σ^*2*^_G_ the genetic variance for a trait_j_, and σ^*2*^_E_ the residual variance. The sum σ^*2*^_G_ + σ^*2*^_E_ corresponds to the overall phenotypic variance (σ^*2*^_P_). The genetic correlation was:


$$\begin{array}{l} cor_{jk}=\;cov_{jk}/sqrt\left({\sigma^{2}}_{gj}x{\sigma^{2}}_{gk}\right) \end{array}$$

with cor_*jk*_ and cov_*jk*_ the genetic correlation and covariance between trait_*j*_ and trait_*k*_, and σ^*2*^_*gj*_ and σ^*2*^_*gk*_ the genetic variances for trait_j_ and trait_k_. The phenotypic correlations were calculated accordingly. Phenotypic correlations were tested for significance using a Pearson’s correlation test. Genetic correlations were considered significant when the interval cor_*jk*_ ± 1.96 × SE_*jk*_ did not include 0.

Similar to our analysis here, linear multitrait models are routinely used for binary traits in practical commercial breeding value evaluations. Both heritability estimates and selection accuracies from linear models were transformed to account for the binary nature of the traits (see details below). Genetic correlations of binary traits estimated using linear models are expected to be unbiased, whereas phenotypic correlations are biased downwards (Mäntysaari et al., 1991).

For mortality traits, the formula of Dempster and Learner (1950) was used to transform the *h*^2^ on the observed scale to the heritability on the underlying liability scale. Phenotypic and residual correlations of Mortality_3_ and Mortality_4_ cannot be estimated because of the limited number of samples in common between the two phenotypes.

### Genome-wide association study

The statistical software DMU was used for a genome-wide association study (**GWAS**), using the leave one chromosome out approach described by Yang et al. (2014). In this approach, the association between genotype and phenotype is carried out using a linear fixed regression model fitting one SNP at a time, but rather than using the full GRM as the random factor for all SNPs, a new partial GRM is calculated every time by removing all the other SNPs on the same chromosome as the SNP in the analysis, calculating a partial GRM on the fly and fitting it as the random effect (Yang et al., 2014). Thus, for GWAS analyses, the following 2-trait model, in which Weight_2_ was always included, was used:


$$\begin{array}{l} Model\;2:\;y_{ij}=\;\mu_{j}+\;bx_{in}+\;a{}_{ij}+\;e_{ij} \end{array}$$

in which *b* is the fixed regression slope, *x*_*in*_ is the genotype of the *n*th SNP marker of an individual *i, a’*_*ij*_ is the random genetic effect explained by the partial GRM, and all the other terms as described before. Weight_2_ was used again to account for potential selection bias. Significance of the SNP effects was determined using Bonferroni correction (0.05/number of SNPs) for genome-wide significance, and as 0.05/(mean number of SNPs per chromosome) for chromosome-wide significance.

The percentage of genetic variance explained by a SNP was calculated following (Lynch and Walsh, 1998), as:


$$\begin{array}{l} {\sigma^{2}}_{SNP}=\;2p\left(1\;\;p\right)b^{2}/{\sigma^{2}}_{G} \end{array}$$

in which *b* is the fixed regression slope from Model 2, *p* is the frequency of the major allele, σ^*2*^_G_ is the total genetic variance explained by the full GRM (from Model 1).

We tested the effect of the presence of a QTL for mortality by specifically modeling the fixed effect of SNP LR664349.1_1710215 (the only SNP associated with all mortality traits) along with the remaining GRM effects in the successive analyses.

### Estimation of genomic breeding values

To assess the influence of a large QTL on breeding values of Mortality_3_, genomic breeding values were estimated in two ways, either excluding or including the top QTL SNP as a fixed effect. We focused only on Weight_2_, Weight_3_, and Mortality_3_ to illustrate the modeling approaches. GEBVs were estimated to solve the mix models with MiX99 (Pitkänen et al., 2022). First, GEBVs of all three traits were estimated using Model 1 and genomic best linear unbiased prediction (GBLUP). In this approach, the GEBVs are the result of modeling the realized genomic relationship matrix.

Second, in the GBLUP+QTL model, also the top SNP was included as a separate fixed factor:


$$\begin{array}{l} Model\;3:\;y_{ij}=\;\mu_{j}+\;QTL_{i}+\;a_{noqtl}+\;e_{ij} \end{array}$$

in which QTL_*i*_ is the individual genotype of the top QTL SNP fitted as a fixed categorical variable, and *a*_*noqtl*_ is the polygenetic genetic effect explained by the partial GRM calculated without the QTL SNP, by first deleting from the genotype data the top QTL SNP in chromosome 6, and then calculating a new partial GRM. To obtain the total GEBV in this approach, the solution of the fixed QTL effect was added to the polygenetic GEBV of an individual obtained via the partial GRM. For both approaches, the (co)variance components needed as input were estimated using the same models in DMU. The models used were the bivariate models, having Weight_2_ always as one of the traits in the analysis.

The results of Mortality_3_ are shown for both GBLUP Model 1 and GBLUP+QTL Model 3, and for body weights, only Model 1 is given because the QTL used in Model 3 was not QTL affecting any weight trait.

### Validation of genomic evaluation with and without explicit QTL modeling

The predictive ability of the GEBVs of Mortality_3_, Weight_2_, and Weight_3_ was validated in two different ways. Both validation steps were run both on GBLUP model and on the GBLUP+QTL model that included the genotype of the top SNP as a fixed effect.

First, a cross-validation approach was used to randomly mask 20% of phenotypes of a trait, and then GEBVs of all fish were estimated. Then, for the masked individuals only, the correlation of their GEBVs to their actual phenotypes was calculated. Accuracy was calculated as the Pearson correlation coefficient between the GEBVs and the observed phenotypic values divided by the square root of the heritability of the trait on the observed scale (Legarra et al., 2008). For Mortality_3_ we also calculated the area under the curve (**AUC**) of the corresponding receiver–operator curve, that is, we assessed the classification performance of the GEBV in predicting the actual mortality phenotypes. For a completely random performance (no classification power) the AUC is 0.5, whereas a perfect classification corresponds to a value of 1. For cross-validation and calculation of AUCs, we ran 1,000 resampling steps, and the means and standard deviation are presented.

When sampling individual fish randomly into the two groups, the reference group and the validation group maintain their close relationship. This is a typical setup when applying genomic selection in commercial aquaculture breeding programs in which all the families are held in the nucleus and their sibs are recorded for hard-to-record traits such as disease resistance, carcass traits, or product quality. Such close relatedness is used to improve the power of genomic selection in commercial breeding, and our estimates of selection accuracy are expected to be higher than if the reference group and the validation group were less related (Fraslin et al., 2022b).

Secondly, we tested the power of the GEBVs of Mortality_3_ to predict the phenotype of Mortality_4_, using all data and the correlation between the GEBVs and phenotypes.

## Results

### Heritability estimates

For all traits, we observed significant heritability estimates. Heritability was 0.44 to 0.58 for Weight_2_ and Weight_3_ (Table 2). Heritability on the observed scale for mortality traits ranged between 0.13 and 0.25, and on the liability scale, it ranged between 0.20 and 0.43 (Table 2).

**Table 2.** Variance components (σ^*2*^_g_: genetic variance; σ^*2*^_e_: environmental variance; σ^*2*^_p_: phenotypic variance) and heritability for recorded traits (*h*^*2*^, both on the observed and on the liability scales for mortality traits)

Trait	σ^*2*^_g_	σ^*2*^_e_	σ^*2*^_p_	*h* ^*2*^ on the observed scale	SE on the observed scale	*h* ^*2*^ on the liability scale	SE on the liability scale
Weight_2_	1,432.14	1,857.12	3,289.26	0.44	0.04		
Weight_3_	15,500.57	13,537.16	29,037.73	0.53	0.05		
Height/Length_3_	1.47E−04	1.05E−04	2.53E−04	0.58	0.05		
Mortality_3_	0.04	0.19	0.23	0.19	0.06	0.33	0.10
Mortality_4_	0.03	0.23	0.26	0.13	0.07	0.20	0.11
Mortality_tot_	0.06	0.17	0.23	0.25	0.06	0.43	0.11

### Phenotypic correlation

The correlation test allowed us to assess whether traits were significantly correlated at the phenotypic level. For the production traits, we observed that Weight_2_ and Weight_3_ were positively and significantly phenotypically correlated (Table 3) and that there was also a positive correlation of Weight_2_ and Weight_3_ with Height/Length_3_. Therefore, all three production-related traits showed a strong positive phenotypic correlation among themselves.

**Table 3.** Phenotypic correlations (lower triangular matrix) and genetic correlations ± standard error (upper triangular matrix)

	Weight_2_	Weight_3_	Height/Length_3_	Mortality_3_	Mortality_4_	Mortality_tot_
Weight_2_	–	0.77 ± 0.05	0.72 ± 0.06	0.16 ± 013	−0.10 ± 0.19	0.05 ± 0.12
Weight_3_	0.63^a^	–	0.81 ± 0.04	−0.20 ± 0.14	0.17 ± 0.20	0.12 ± 0.13
Height/Length_3_	0.5^a^	0.78^a^	–	−0.19 ± 0.14	0.05 ± 0.21	−0.01 ± 0.14
Mortality_3_	0.22^a^	−0.08^d^	−0.08^d^	–	0.90 ± 0.18	0.99 ± 0.04
Mortality_4_	−0.03^d^	0.17^a^	0.08^c^	^NA^	–	1
Mortality_tot_	0.11^b^	0.20^a^	0.08^d^	0.5^a^	1	–

^a^ *P*-value < 0.001.

^b^ *P*-value < 0.01 and *P*-value > 0.001.

^c^ *P*-value < 0.05 and *P*-value > 0.01.

^d^Not significant.

NA, not available.

Testing for a correlation between weight traits and mortality, we observed that Weight_2_ was positively and significantly phenotypically correlated with Mortality_3,_ whereas the correlation between Weight_2_ and Mortality_4_ was not significant. In contrast, the correlation between Weight_2_ and Mortality_tot_ was once again positive and significant. We also observed that Weight_3_ was not significantly correlated with Mortality_3_, but it was positive and significant with Mortality_4_ and with Mortality_tot_. In all cases where the correlation test was significant, the phenotypic correlation between weight and mortality was positive, indicating that at the phenotypic level, heavier fish had a higher mortality.

For Height/Length_3_, we did not observe a significant correlation with Mortality_3_, though we did observe a positive and significant correlation with Mortality_4_, and then again, we did not observe a significant correlation with Mortality_tot_.

Finally, all mortality traits were strongly correlated. Mortality_3_ was positively and significantly correlated with Mortality_tot_, and Mortality_4_ and Mortality_tot_ have a correlation of 1. Mortality_3_ and Mortality_4_ could not have a direct phenotypic correlation because the samples that reached Mortality_4_ were all alive at Mortality_3_, making it impossible to calculate a phenotypic correlation between the two traits.

### Genetic correlation

Genetic correlations broadly followed the phenotypic correlations (Table 3), but all the genetic correlations of mortality traits with the weight traits had very large standard errors, thus indicating that the correlations are not significant. On the other hand, Weight_2_ was positively and significantly genetically correlated with Weight_3_ and Height/Length_3_ (Table 3). Weight_3_ was also positively and significantly genetically correlated with Height/Length_3_. Mortality_3_ was significantly genetically correlated with Mortality_4_ (0.90) and Mortality_tot_ (0.99). The correlation between Mortality_4_ and Mortality_tot_ was estimated as 1 with no standard error estimation. These high positive correlations imply that mortality has similar genetic determination across ages.

### GWAS results

**Figure 1.**
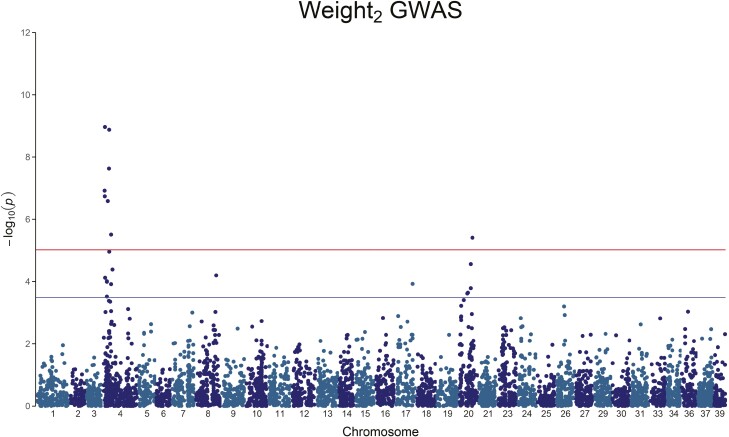
Manhattan plot of the *−*log_10_(*P*-value) of Weight_2_. A clear, genome-wide significant, QTL peak is visible on Chromosome 4, with a second peak on chromosome 20.

**Figure 2.**
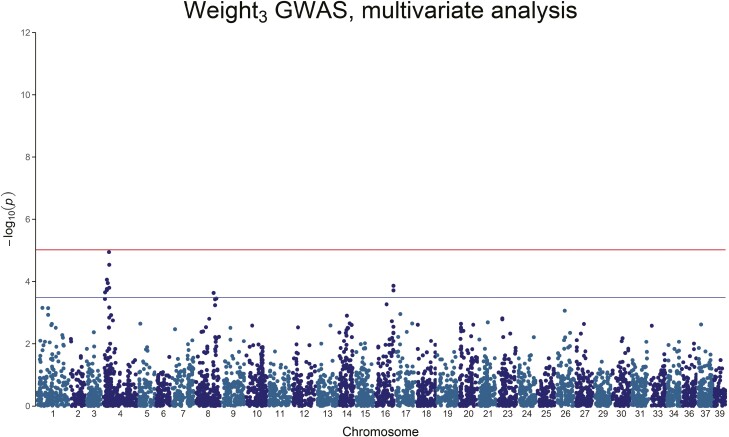
Manhattan plot of the *−*log_10_(*P*-value) of Weight_3_. A chromosome-wide significant QTL peak is visible on Chromosome 4, but the peak does not reach genome-wide significance.

**Figure 3.**
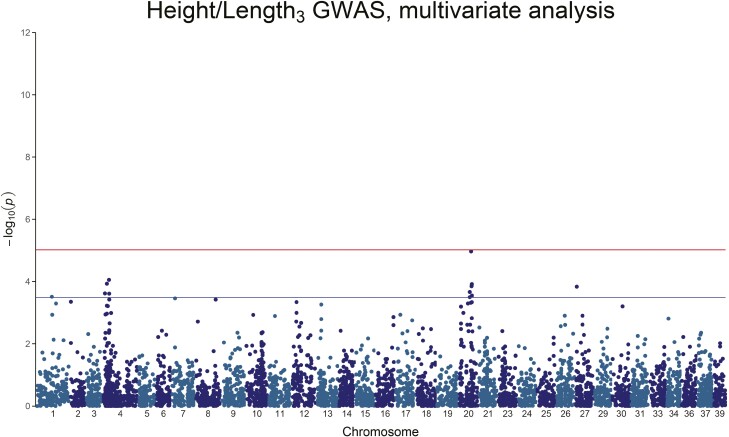
Manhattan plot of the *−*log_10_(*P*-value) of Height/Length_3_. A chromosome-wide significant QTL peak is visible on Chromosome 4, with a second chromosome-wide peak on chromosome 20. Neither peak reaches genome-wide significance.

**Figure 4.**
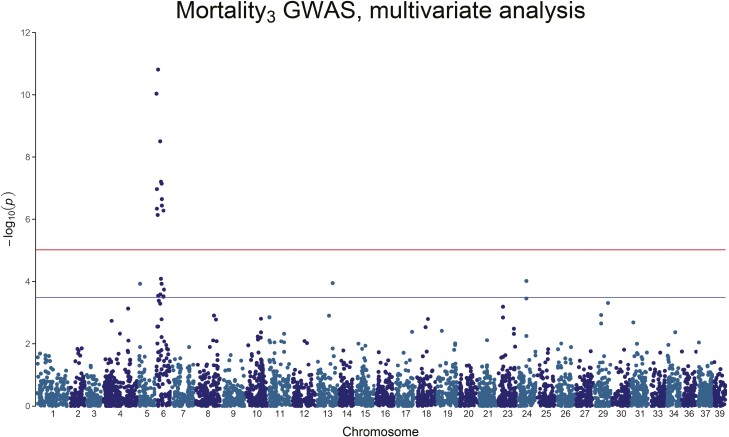
Manhattan plot of the *−*log_10_(*P*-value) of Mortality_3_. A clear, genome-wide significant, QTL peak is visible on Chromosome 6.

**Figure 5.**
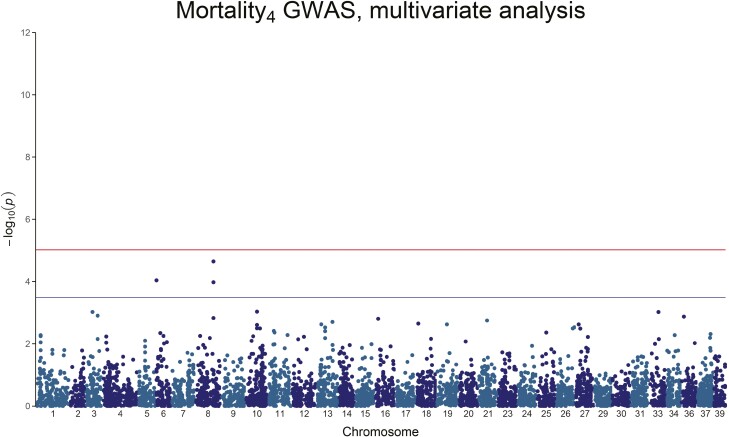
Manhattan plot of the *−*log_10_(*P*-value) of Mortality_4_. No QTL peaks are visible.

**Figure 6.**
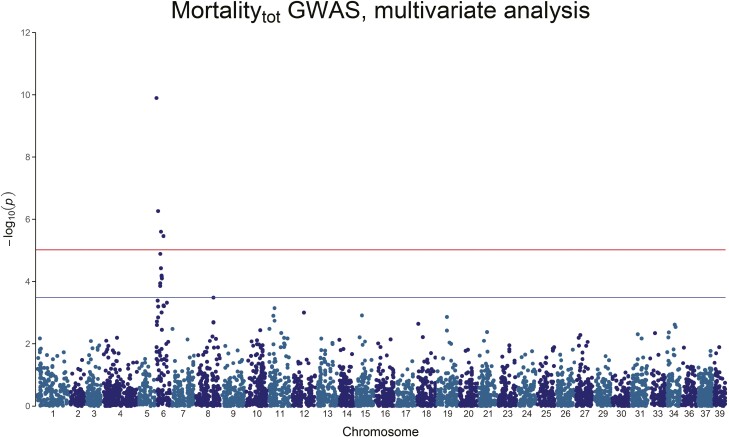
Manhattan plot of the *−*log_10_(*P*-value) of Mortality_tot_. A clear, genome-wide significant, QTL peak is visible on Chromosome 6.

Multiple traits and DNA variants showed a genome-wide or chromosome-wide association (Table 4 and Figures 1–6, the linkage disequilibrium between markers is presented in Supplementary Figure S1). Weight_2_ showed two genome-wide significant peaks: one, significant, on chromosome 4, and a second on chromosome 20 (Figure 1 and Table 4). The amount of genetic variance explained by these genome-wide SNPs ranges between 2.76% and 5.40%. Weight_3_ does not have any genome-wide significant SNPs, but we observe chromosome-wide significant peaks on chromosome 4, chromosome 8, and chromosome 16, with the genetic variance explained by any of these SNPs ranging between 1.77% and 6.30% (Figure 2 and Table 4).

**Table 4.** Genome-wide significant SNPs associated with traits. *P*-values shown are Bonferroni corrected, unless otherwise indicated. SNP names reflect the internal nomenclature of our RAD sequencing, chromosome and base-pair position are based on the assembly of De-Kayne et al. (2022)

SNP name	chr	bp	Beta	SE	Sample size	*t*	*P*	−log_10_(*p*)	MAF	% genetic variance explained
Weight_2_										
LR664347.1_2201425	4	2,201,425	15.73	2.95	1,095	5.33	1.21E−07	6.92	0.38	4.05
LR664347.1_2693802	4	2,693,802	13.68	2.61	1,130	5.25	1.83 E−07	6.74	0.47	3.26
LR664347.1_3146138	4	3,146,138	−15.67	2.55	1,108	6.15	1.09 E−09	8.96	0.49	4.28
LR664347.1_10995075	4	10,995,075	13.96	2.69	1,120	5.18	2.60 E−07	6.58	0.28	2.76
LR664347.1_14268077	4	14,268,077	15.88	2.82	1,185	5.62	2.36 E−08	7.63	0.30	3.71
LR664347.1_14836448	4	14,836,448	14.53	2.38	1,127	6.11	1.33 E−09	8.88	0.34	3.31
LR664347.1_20091990	4	20,091,990	−18.01	3.84	1,115	4.69	3.12 E−06	5.51	0.39	5.40
LR664363.1_38855216	20	38,855,216	15.01	3.24	1,166	4.64	3.92 E−06	5.41	0.48	3.93
Weight_3_										
LR664347.1_3568737	4	3,568,737	−52.35	14.07	498	3.72	2.21 E−04	3.65	0.30	3.72
LR664347.1_8489919	4	8,489,919	−62.48	16.61	518	3.76	1.88 E−04	3.73	0.42	6.12
LR664347.1_8489934	4	8,489,934	−63.02	16.67	546	3.78	1.73 E−04	3.76	0.44	6.30
LR664347.1_8489940	4	8,489,940	−60.67	15.35	544	3.95	8.76 E−05	4.06	0.42	5.80
LR664347.1_10995075	4	10,995,075	40.77	10.48	527	3.89	1.12 E−04	3.95	0.28	2.17
LR664347.1_14268077	4	14,268,077	46.80	10.56	562	4.43	1.13 E−05	4.95	0.30	2.98
LR664347.1_14836448	4	14,836,448	34.93	9.18	518	3.81	1.59 E−04	3.80	0.34	1.77
LR664347.1_14962376	4	14,962,376	54.65	12.95	521	4.22	2.90 E−05	4.54	0.42	4.70
LR664351.1_49728274	8	49,728,274	−51.05	13.77	540	3.71	2.31 E−04	3.64	0.24	3.06
LR664359.1_50667396	16	50,667,396	41.52	10.80	521	3.84	1.37 E−04	3.86	0.27	2.18
LR664359.1_50667662	16	50,667,662	40.96	10.90	511	3.76	1.92 E−04	3.72	0.26	2.10
Height/Length_3_										
LR664344.1_44836701	1	44,836,701	0.00	0.00	539	3.63	3.07 E−04	3.51	0.31	2.08
LR664347.1_3146138	4	3,146,138	0.00	0.00	513	3.70	2.40 E−04	3.62	0.49	1.94
LR664347.1_8489940	4	8,489,940	−0.01	0.00	544	3.88	1.18 E−04	3.93	0.42	5.48
LR664347.1_14268077	4	14,268,077	0.00	0.00	562	3.95	8.86 E−05	4.05	0.30	2.32
LR664347.1_14836448	4	14,836,448	0.00	0.00	518	3.69	2.44 E−04	3.61	0.34	1.65
LR664363.1_31261053	20	31,261,053	0.00	0.00	543	3.72	2.16 E−04	3.67	0.30	2.30
LR664363.1_31261188	20	31,261,188	0.00	0.00	521	3.62	3.18 E−04	3.50	0.30	2.01
LR664363.1_34941325	20	34,941,325	0.00	0.00	514	4.45	1.07 E−05	4.97	0.26	2.95
LR664363.1_36174451	20	36,174,451	0.00	0.00	538	3.83	1.41 E−04	3.85	0.40	2.60
LR664363.1_36909591	20	36,909,591	0.00	0.00	507	3.87	1.22 E−04	3.91	0.28	2.57
LR664363.1_37414088	20	37,414,088	0.00	0.00	553	3.87	1.22 E−04	3.91	0.30	2.51
LR664363.1_37414306	20	37,414,306	0.00	0.00	541	3.66	2.81 E−04	3.55	0.41	2.48
LR664370.1_1484511	27	1,484,511	0.01	0.00	525	3.83	1.46 E−04	3.84	0.21	3.19
Mortality_3_										
LR664349.1_1710215	6	1,710,215	−0.18	0.03	725	6.58	9.22 E−11	10.04	0.39	36.21
LR664349.1_2924390	6	2,924,390	0.16	0.03	673	5.37	1.08 E−07	6.97	0.30	22.67
LR664349.1_2924638	6	2,924,638	0.15	0.03	683	5.09	4.57 E−07	6.34	0.30	20.32
LR664349.1_5024842	6	5,024,842	0.15	0.03	759	5.00	7.30 E−07	6.14	0.43	26.39
LR664349.1_6154414	6	6,154,414	−0.22	0.03	731	6.85	1.55 E−11	10.81	0.38	51.34
LR664349.1_11957954	6	11,957,954	−0.21	0.03	728	6.00	3.12 E−09	8.51	0.23	33.92
LR664349.1_14128348	6	14,128,348	0.15	0.03	710	5.47	6.21 E−08	7.21	0.44	23.83
LR664349.1_16037751	6	16,037,751	0.15	0.03	733	5.44	7.14 E−08	7.15	0.44	25.66
LR664349.1_16570835	6	16,570,835	−0.19	0.04	743	5.23	2.25 E−07	6.65	0.23	27.53
LR664349.1_16619105	6	16,619,105	−0.18	0.04	710	5.14	3.62 E−07	6.44	0.19	23.52
LR664349.1_21146341	6	21,146,341	0.15	0.03	726	5.06	5.27 E−07	6.28	0.35	24.11
Mortality_4_										
LR664349.1_1710215	6	1,710,215	−0.16	0.04	419	3.95	9.18 E−05	4.04	0.39	18.39
LR664351.1_49261152	8	49,261,152	0.22	0.05	431	4.28	2.26 E−05	4.65	0.26	29.97
LR664351.1_49261323	8	49,261,323	0.20	0.05	449	3.91	1.06 E−04	3.98	0.27	24.73
Mortality_tot_										
LR664349.1_1710215	6	1,710,215	−0.20	0.03	631	6.54	1.28 E−10	9.89	0.39	15.82
LR664349.1_6154414	6	6,154,414	−0.18	0.03	637	5.06	5.46 E−07	6.26	0.38	12.40
LR664349.1_14128348	6	14,128,348	0.14	0.03	624	4.75	2.51 E−06	5.60	0.44	7.65
LR664349.1_21146341	6	21,146,341	0.15	0.03	635	4.68	3.48 E−06	5.46	0.35	9.13

SNP name: provisional SNP id; chr: chromosome; bp; base pair; beta: regression coefficient; SE: regression coefficient’ standard error; Sample size: number of sample in each linear model; *t*: *t*-value; *P*: *P*-value; −log_10_(*P*): −log_10_(*P*-value); MAF: minor allele frequency; % genetic variance explained: genetic variance explained by the SNP for the trait.

Height/Length_3_ also only has chromosome-wide level association, with peaks on chromosome 1, chromosome 4, chromosome 20, and chromosome 27, with the genetic variance explained by any of these SNPs ranging between 1.65% and 3.19% (Figure 3 and Table 4). All body weight traits involve chromosome 4, and, to a smaller extent, chromosome 20.

For Mortality_3_, there is a strong genome-wide significant peak on chromosome 6, with 20.3% to 51.3% of the genetic variance explained by the significant SNPs (Figure 4 and Table 4). Mortality_4_ on the other hand does not show any genome-wide significant association, but only 3 chromosome-wide significant SNPs on chromosome 6 and chromosome 8, explaining 18.4% to 30.0% of the genetic variance (Figure 5 and Table 4). Mortality_tot_ shows one single genome-wide peak on chromosome 6 (the genome-wide significant SNP for Mortality_4_ on Chromosome 6 is also genome-wide significant for Mortality_3_ and Mortality_tot_), explaining 7.65% to 15.82% of the genetic variance (Figure 6 and Table 4). In all cases, the mortality traits involve chromosome 6, with no overlap with the QTL found for the weight traits.

**Figure 7.**
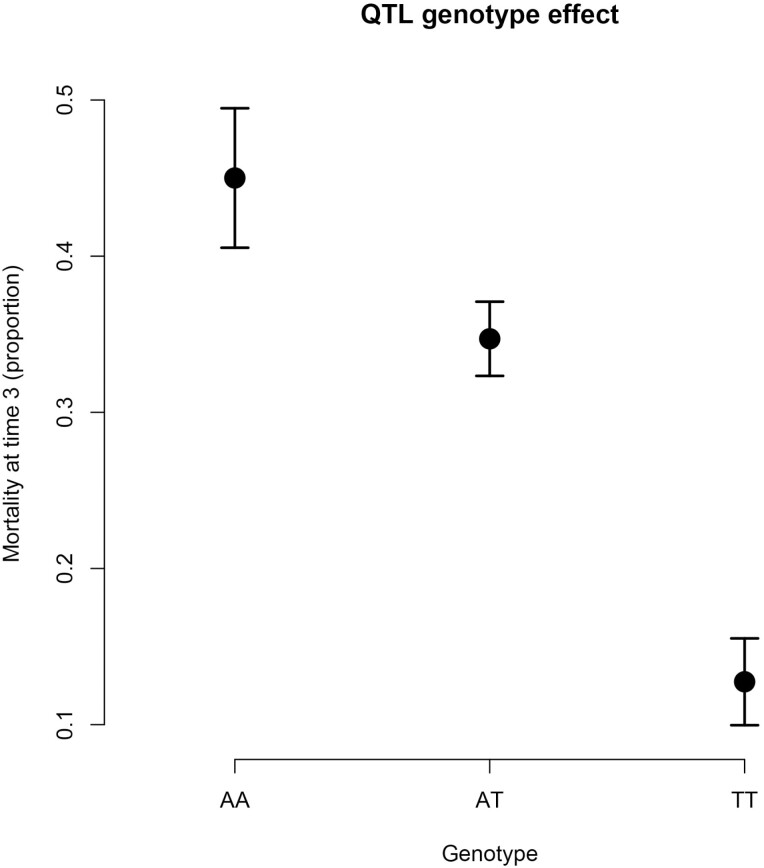
Allele substitution effects for the top SNP associated with Mortality at time 3. The pattern is consistent with an additive effect of each allele substitution.

For Mortality_3_, the regression slope of the most associated locus against mortality (*b* in QTL+GBLUP model) is −0.18. When the top SNP for Mortality_3_ is fitted as a categorical fixed effect, the result does not indicate any signs of dominance (Figure 7), and the difference between the extreme genotypes is 32% in mortality between the two homozygote genotypes.

### Validation of genomic evaluation with GBLUP and GBLUP+QTL

**Figure 8.**
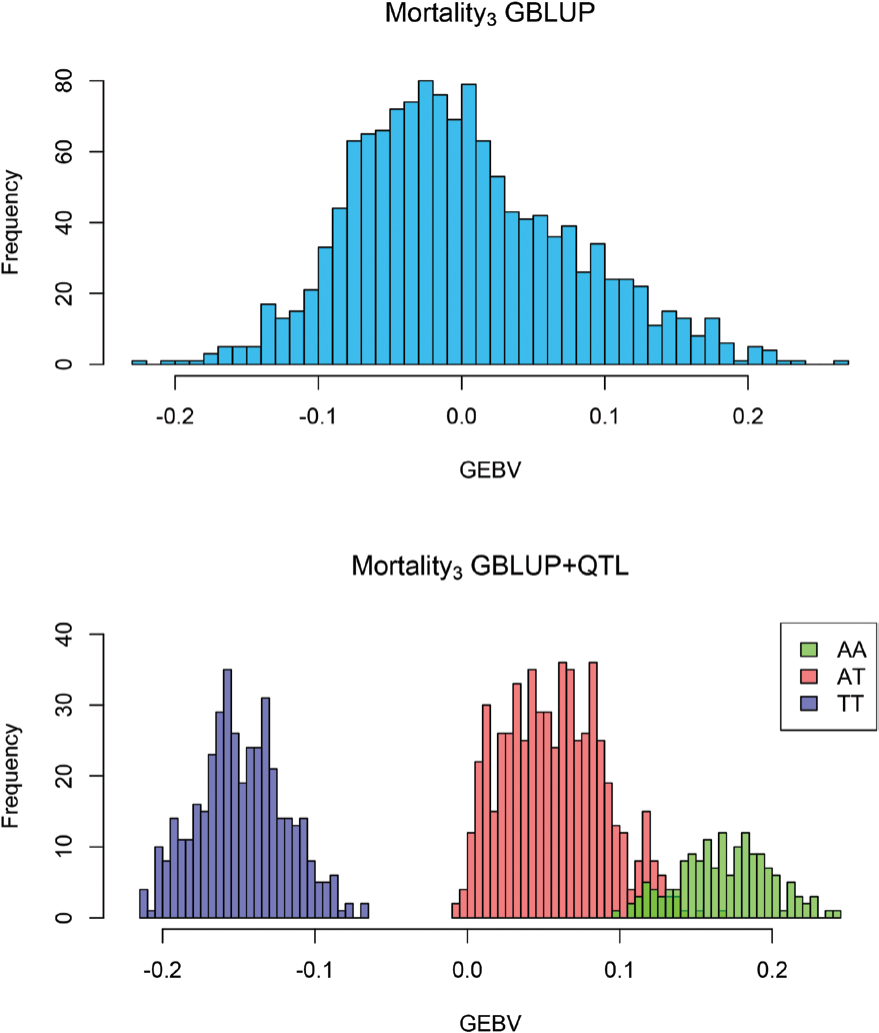
Genomic breeding values estimated with GBLUP and GBLUP+QTL models. In GBLUP+QTL model, the top SNP effect with genotypes AA, AT and TT were fitted as a fixed effect.

Genomic breeding values of Mortality_3_ from the GBLUP and GBLUP+QTL models are shown in Figure 8. In GBLUP model, the GEBVs range between −0.22 and 0.26, and in the GBLUP+QTL model between −0.21 and 0.24.

**Figure 9:**
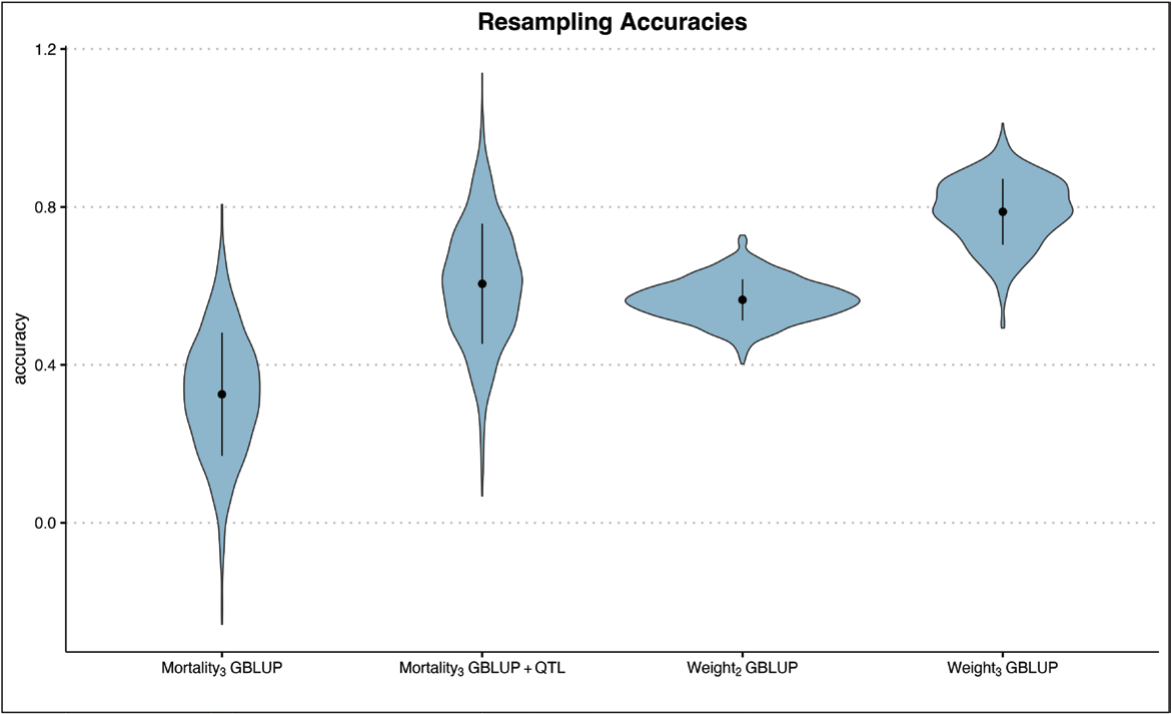
Violin plot of cross-validation accuracies of mortality and weight traits in GBLUP and GBLUP+QTL models. Dark area shows the distribution of accuracies in 1,000 resampling runs.

**Figure 10.**
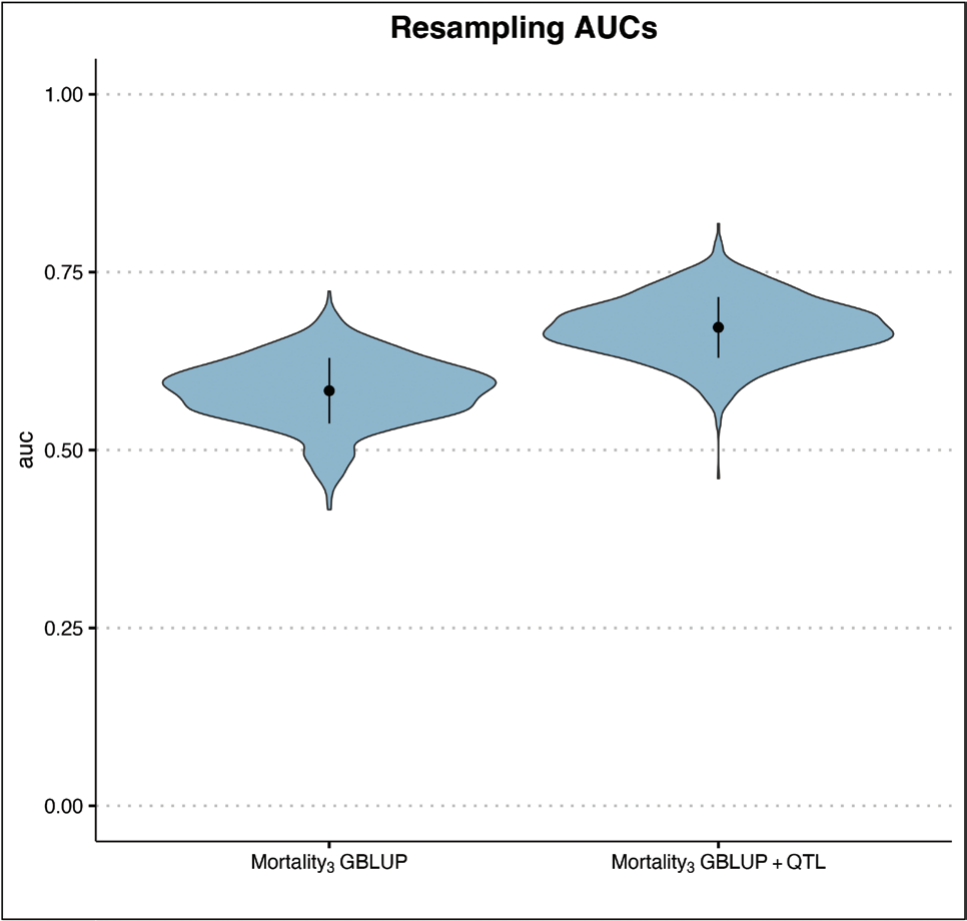
Violin plot of the area under the curve (**AUC**) of mortality at time 3 in GBLUP and GBLUP+QTL models. Dark area shows the distribution of AUC in 1,000 resampling runs.

In the cross-validation, for the GBLUP model we observe a mean accuracy of 0.33 for Mortality_3_ (Figure 9). For the GBLUP+QTL model, the accuracy for mortality at time 3 is much higher, 0.61 (Figure 9). When calculating the AUC values, we observe a mean of 0.58 AUC for the GBLUP model, whereas the GBLUP+QTL resulted in a 0.67 mean AUC (Figure 10).

As a comparison, for Weight_2_ the mean accuracy was 0.56, and for Weight_3_ the mean accuracy was 0.79 (both GBLUP models).

The cross-validation accuracies showed large variation, especially in mortality traits (Figure 9). For binary traits such as mortality, the varying incidence of surviving fish in the reference group (or validation group) due to random sampling of the fish into the two groups has a major impact on the accuracy. Moreover, due to the random sampling, the relationships between reference and validation groups may change, impacting accuracies.

When we test the predictive ability of GEBVs of Mortality_3_ for Mortality_4_, we observe a positive correlation between Mortality_3_ GEBV with Mortality_4_ phenotype, of 0.18 for the GBLUP model, and of 0.22 for the GBLUP+QTL model (Table 5). When we assess the predictive ability of Mortality_3_ GEBV for Mortality_4_ GEBV, calculated together using one single multivariate model, we observe a positive correlation between Mortality_3_ GEBV and Mortality_4_ GEBV, of 0.77 for GBLUP model, and 0.91 for the GBLUP+QTL model (Table 5).

**Table 5.** Correlations of GEBVs of mortality 3 with mortality at time 4

	Mortality_3_	Mortality_4_	GBLUP	GBLUP+QTL
Correlation	GEBV	GEBV	0.77	0.91
Correlation	GEBV	Phenotype	0.18	0.22

## Discussion

### Feasibility of using NGS for genomic selection

Our first goal, the assessment of the use of NGS for genomic selection in European whitefish was broadly successful. We were able to capture a total of 5,242 SNP markers, an average of 154 SNPs for each chromosome, with an average SNP spacing of 351 kilobases. This result provided ­sufficient information for the estimation of the pairwise genomic relationship between fish, and thus the GRM. Recent studies based on cross-validations in multiple aquaculture species and different traits imply that around 2,000 to 7,000 SNPs are needed for accurate genomic evaluation (Kriaridou et al., 2020; Fraslin et al., 2022b). The use of a GRM is obviously key for modern genomic evaluations, and our NGS approach allowed us to both fulfill this requirement for genomic selection and to extend our analysis to a GWAS. Yet, for more fine-grained QTL mapping, a higher-density SNP panel would be needed.

NGS offers great potential for aquaculture, especially for those species that do not have prior genomic information and no off-the-shelf SNP chips (Robledo et al., 2017). NGS can be used to identify enough markers to calculate the genomic relationship matrix, bypassing the need to use a pedigree record. The ability to produce genome-wide SNPs is especially valuable because it allows to capture much of the genetic variance, irrespective of what is known of the actual genetic architecture of the target trait(s). The successful use of NGS for this goal has been observed, for instance, in olive flounder (using 12,712 SNPs, Shao et al., 2015), gilthead sea bream (using 12,085 SNPs, Palaiokostas et al., 2016), barramundi (using 3,321 SNPs, Wang et al., 2015), bighead carp (using 323 SNPs, Fu et al., 2016), scallop (using 2,364 SNPs, Dou et al., 2016), Japanese sea cucumber (using 5,517 SNPs, Tian et al., 2015), and abalone (using 3,717 SNPs, Ren et al., 2016).

The most obvious limitations of NGS are that it is much more computationally and labor intensive than SNP chips, especially in the absence of a reference genome, and that a good overlap between SNP sets between different genotyping runs is never guaranteed, making comparing or combining datasets complex. To obviate these two drawbacks, once a set of SNPs of the desired genome-wide density has been achieved, and a reliable reference genome is available, it is possible to develop custom SNP arrays and/or genotyping panels.

### Genetic characteristics of mortality to *Saprolegnia*

To our knowledge, there are no previous reports on the genomic characterization of mortality for *Saprolegnia* in fish. Our results show that for each mortality trait, *h*^*2*^ was low to moderate on the observed scale (0.13 to 0.25), and *h*^*2*^ on the underlying liability scale showed a substantial increase (0.20 to 0.43), suggesting that the genetic component for the mortality traits is actually bigger than what can be directly observed. These estimates are clearly higher than the *h*^*2*^ estimates of general mortality whose causes are unknown (*h*^*2*^ range 0.08 to 0.17 on the liability scale in rainbow trout, Vehviläinen et al., 2008; *h*^*2*^ range 0.07 to 0.20 on the liability scale, also in rainbow trout, Vehviläinen et al., 2010).

We found a major QTL for survival to infection to this oomycete in whitefish, with the SNPs involved in the main QTL each explaining an average of 21% of the genetic variance for mortality. This was in strong contrast to the polygenetic nature of body weight. Immunity to infection often shows a monogenic or oligogenic architecture in fish (e.g., Fuji et al., 2007; Moen et al., 2009; Houston et al., 2010; Calboli et al., 2022; Fraslin et al., 2022a). This may be because immunity can have a simple mechanism that changes the level of resistance, such as the case of infectious pancreas necrosis in European Atlantic salmon, which is almost completely determined by the *nedd-8* locus (Pavelin et al., 2021), and potentially because aquaculture species have been only recently domesticated and thus QTLs of large effects have not been brought to fixation across commercial stocks. In our study, the top SNP shows an additive pattern of effect with 32% difference in mortality between the most resistant homozygote genotype and the most susceptible homozygote genotype, providing a valuable target for selection in aquaculture settings. This is the first major *Saprolegnia* infection experienced by this fish stock, but the fungus causes major economic losses and reduces fish health in aquaculture.

Based on the annotation available on National Center for Biotechnology Information (NCBI), the QTL on chromosome 6 overlays six different putative genes (Supplementary Table S1). Blasting the amino acid sequences we identified the closest homologs in other species. Only for two of the putative genes the best homologs match has an amino acid sequence of a matching length, the tRNA methyltransferase and the breast cancer type 2 susceptibility protein, whereas the other proteins in the *Coregonus lavaretus* ‘Balchen’ assembly match only a fragment of the possible homologue proteins. Both the tRNA methyltransferase and the breast cancer type 2 susceptibility protein are involved in tumorigenesis in humans, which might suggest they play a role in the immunity to infections in European whitefish, though these results are observational, and substantial more work would be needed to infer any functional effect—such as the functional genetic approach used by Palvelin et al. (2021) to show that is *nedd-8*, rather than the adjacent *cdh1* locus controlling resistance to IPNV infection.

One of the important considerations stemming from our work is the importance of choosing the most informative time-period to collect mortality data in a breeding program. Because the number of fish surviving over time decreases over time, our ability to calculate precise GEBVs for all traits, not just mortality, decreases over time, as the sample size shrinks. In our data, we observed a strong genetic correlation between the mortality traits over time (Table 3), and that the genetics of survival to *Saprolegnia* is nearly identical at all time points. Thus, our data suggest that using an early mortality to *Saprolegnia* measure would provide a robust proxy for all mortality to *Saprolegnia* at all times during the breeding program. Mortality data collected before the maturity will allow to estimate GEBVs and make selection well in advance before the fish are spawning.

Importantly, while the genetic correlation between size-based traits and mortality traits is low with large standard errors, the phenotypic correlations are either not significant, or positive and significant, suggesting that phenotypically larger fish at any time point are more likely to have died by the time the next mortality census is carried out. The low genetic correlations mean that both weight and mortality can be improved simultaneously, yet care should be taken to track potentially correlated genetic changes in multiple traits.

In addition, the phenotypic correlation between weight and survival indicates that the phenotypically the smallest fish tend to die during growth, creating a selection bias in the data. When such selection bias occurs, it needs to be taken into account by including the pre-selected trait(s) recorded from all the individuals in a multivariate animal model (Henderson, 1975; Ouweltjes et al., 1988). Hence, in the estimation of genetic parameters and GEBVs as well as in GWAS, we always included the initial body Weight_2_ as a trait in all (multivariate) analyses.

### Genetic characteristics of body size

Heritability for body weight was moderate (0.44 to 0.53) and of similar magnitude to what has been observed previously for this species (Kause et al., 2011), yet these estimates are at the higher end of the range typically observed for salmonids (Carlson and Seamons, 2008; Kause et al., 2022). It is interesting to notice that for body weight we also observe a QTL of high statistical significance, but for this trait, the amount of genetic variance explained by the SNPs included in the QTL is much smaller than for mortality: the SNPs involved in the main QTL explain an average of 3% genetic variance for body weight. The main QTL for body weight maps on the same chromosome, but roughly 25 megabases apart from a previously reported sex-determining QTL in fish in the same species complex (De-Kayne et al., 2022). In our GWAS results, this region is not associated with any fish trait we recorded.

### Genomic selection for mortality to *Saprolegnia*

Our results highlight that, irrespective of any other consideration, the benefit of testing for the presence of QTLs before estimating genomic breeding values is worth the extra effort in breeding settings. The inclusion of the QTL in genomic breeding value evaluation increased the accuracy of GEBVs, with a clear change in GEBV distribution pattern matching the actual QTL genotype. Estimation of GEBVs based on genotype data would require only a minimum amount of extra computational effort to test for QTLs, because the required phenotypic data would need to be ascertained in any case, with obvious benefits in terms of potentially increased selection accuracy due to the more detailed modeling of the mode of inheritance.

The assumption of genomic evaluation with a genomic relationship matrix (GBLUP) is that SNP effects are small and normally distributed, but these assumptions do not necessarily hold in practice. When these assumptions do not hold, other sophisticated statistical approaches have been developed. Examples of these approaches are Bayesian methods that can model non-normal distribution of SNP effects (Habier et al., 2011), weighted single step in which SNP effects are used to weight markers in GEBV estimation (Zhang et al., 2016), and trait-specific weighted GRM matrices (Fragomeni et al., 2017). The major limitation of these approaches is that currently, these methods cannot be effectively implemented in multivariate analyses where potentially many tens of traits are analyzed together, a ­practice that is standard in routine breeding value evaluation. For strongly oligogenic traits, genomic evaluation with MAS offers a practical solution from a breeding standpoint, preserving the ability to use the multivariate analysis when the focus is purely on breeding.

For strongly oligogenic traits, such as for *Saprolegnia* resistance here, the use of GBLUP+QTL is currently in fact an appealing approach. For instance, we validated the GEBV prediction improvement using a cross-validation approach. We observed an average of 0.33 in the accuracy for mortality calculated using the GBLUP model, but the accuracy increased to a mean of 0.61 for the GBLUP+QTL model, almost a doubling of the predictive accuracy. Additionally, when we used the AUC approach, to account for the fact that mortality is binary trait, we observed that the mean accuracy for the GBLUP+QTL (0.67) is not only higher than the mean accuracy for the GBLUP (0.59), but it is very close to the maximum theoretical accuracy we can expect for this dataset, which is 0.7 (Wray et al., 2010). Furthermore, the ability of Mortality_3_ GEBVs estimated with the GBLUP and GBLUP+QTL models to predict the Mortality_4_ phenotype improves from 0.46 in the GBLUP model to 0.61 for the GBLUP+QTL. All these observations indicate that the GBLUP+QTL improves our predictive ability compared to the simpler GBLUP model. Of course, these results are currently limited to the present subset of data, and the effect of the QTL estimated in a single fish group. The results would have to be validated over different generations of the breeding program to assess the overall, general effect of the GEBVs and QTL on survival to *Saprolegnia*.

Overall, our results indicate the value of using NGS techniques for genomic selection on European whitefish aquaculture, and highlight the benefit that genome-wide markers offer, not just for genomic selection, but for exploring the genetic architecture of traits under selection, and to increase the precision of the estimation of GEBVs. Additionally, we identified the presence of a major QTL for the resistance to *Saprolegnia*, a common disease affecting this and many other fish species in aquaculture, indicating that there are opportunities to use genomic selection to improve resistance to *Saprolegnia*.

## Supplementary Material

skad333_suppl_Supplementary_Table_S1

## Abbreviations:

AUC: area under the curve
ddRAD: double digest restriction site associated DNA
GRM: genetic relationship matrix;
h2: heritability
GEBV: genomic estimated breeding value
GWAS: Genome-Wide Association Study
MAS: marker-assisted selection
NGS: next generation sequencing
QTL: quantitative trait loci
