# Supplementary figures and images for: Genomic selection for survival under naturally occurring Saprolegnia oomycete infection in farmed European whitefish Coregonus lavaretus
Source: J Anim Sci. 2023 Oct 1;101:skad333. doi: 10.1093/jas/skad333 (PMC10583997; doi:10.1093/jas/skad333)

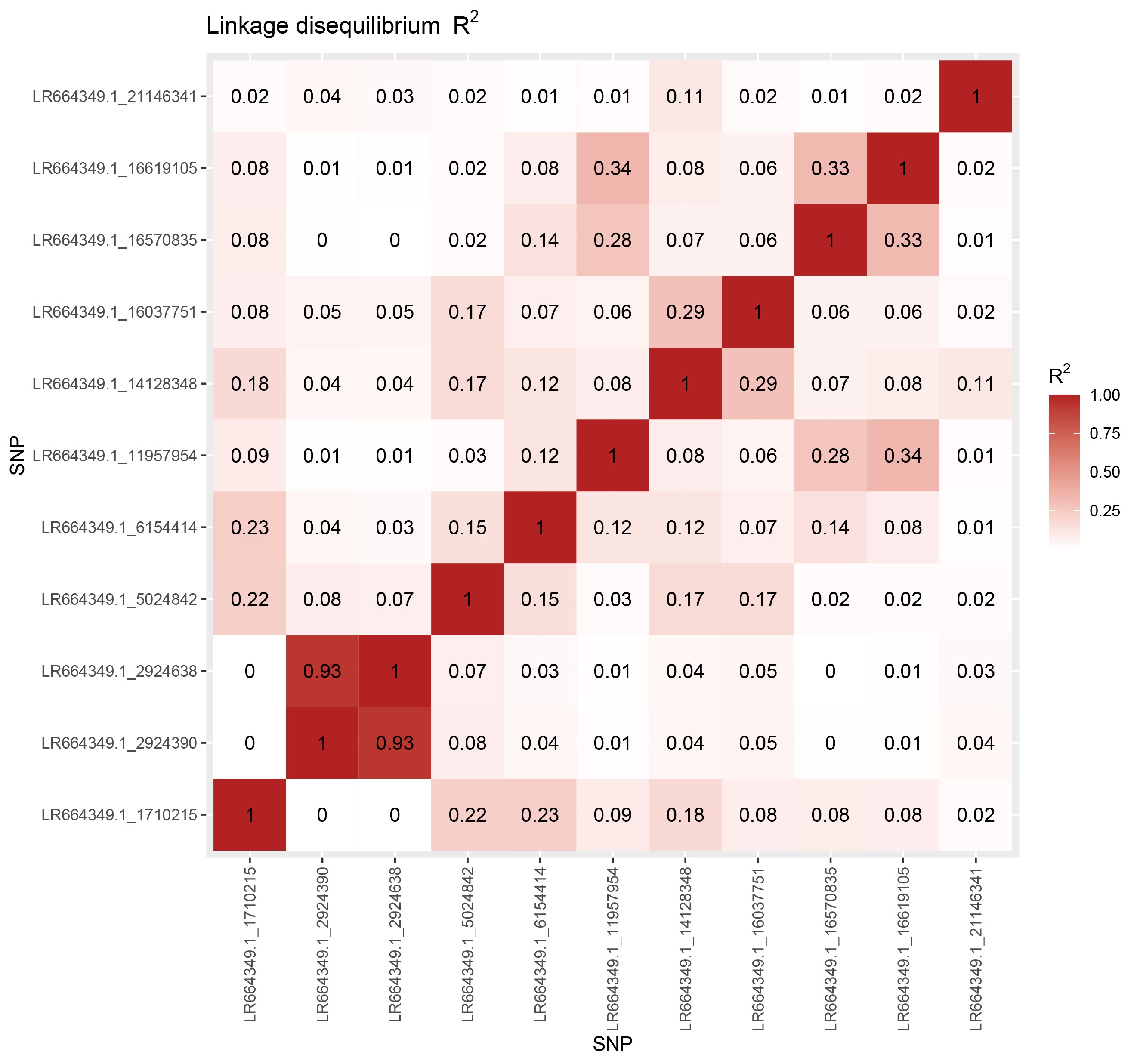

Supplement: skad333_suppl_Supplementary_Table_S1 [file skad333_suppl_supplementary_table_s1.jpeg]
